# Evaluation of Long-Read Genome Sequencing for Genomic Profiling of Myeloid Cancers

**DOI:** 10.1016/j.jmoldx.2025.09.001

**Published:** 2025-09-26

**Authors:** Haley J. Abel, Mohamed Mahgoub, Nidhi Davarapalli, Rohan Kodgule, Christopher A. Miller, Robert S. Fulton, Catrina Fronick, Christopher Markovic, Sharon Heath, Jacqueline E. Payton, Meagan A. Jacoby, Daniel C. Link, Matthew J. Walter, Eric J. Duncavage, Timothy J. Ley, David H. Spencer

**Affiliations:** ∗ Division of Oncology, Department of Medicine, Washington University School of Medicine, St. Louis, Missouri; † Department of Pathology and Immunology, Washington University School of Medicine, St. Louis, Missouri; ‡ McDonnell Genome Institute, Washington University School of Medicine, St. Louis, Missouri

## Abstract

Whole-genome sequencing (WGS) is a comprehensive approach for the genomic evaluation of acute myeloid leukemia (AML) and myelodysplastic syndromes (MDS). We recently described a streamlined tumor-only WGS assay (ChromoSeq) that uses Illumina short-read sequencing with targeted analysis to detect the full range of clinically relevant somatic mutations. Here we sought to determine the performance of this targeted analysis approach using long-read sequencing data from Oxford Nanopore Technologies and Pacific Biosciences. Samples from 26 patients with AML and MDS were sequenced to a mean of 52× coverage. Head-to-head comparison of reportable somatic variants to standard WGS revealed more than 96% recall and 91% precision for single nucleotide variants for both long-read platforms. Performance was lower for insertion/deletions (66% recall and 42% precision), especially in regions with few phased reads that facilitate accurate variant detection. The long-read platforms were 95% accurate for copy number calls, and they detected all recurrent structural variants with no false-positive findings. In addition, long reads properly identified intronic insertions near repetitive elements that were incorrectly identified as interchromosomal structural rearrangements by standard WGS. These results indicate that targeted, tumor-only analysis of long-read sequence data is a feasible approach for the genomic evaluation of myeloid cancers, and they show the utility of incorporating variants discovered via long-read sequencing to improve variant interpretation in short-read WGS.

Genomic evaluation is essential for diagnostic classification and risk assessment of patients with myeloid malignancies and is based on the presence of recurrent cytogenetic abnormalities and somatic gene mutations.^1^ These mutations are usually identified via complex testing algorithms that can involve multiple clinical assays, including G-banded karyotyping, fluorescence *in situ* hybridization, and targeted sequencing of selected genes. We recently described a comprehensive genomic profiling assay, ChromoSeq, for patients with acute myeloid leukemia (AML) and myelodysplastic syndromes (MDS) that uses whole-genome sequencing (WGS) on the Illumina (San Diego, CA) sequencing platform.^2^ This approach combines rapid WGS library preparation with targeted analysis that is focused on large copy number alterations, recurrent structural variants (SVs) (eg, translocations), and small variants in selected, recurrently mutated genes. Comparison of ChromoSeq results versus results from conventional multimodality testing showed that it was highly sensitive and specific for the full spectrum of clinically relevant mutations in patients with AML and MDS and that the results can be directly used in established risk stratification systems.

WGS-based testing strategies have several advantages over conventional testing methods that are of benefit to clinical laboratories, treating physicians, and patients. These include the requirement for DNA as opposed to cultured cells, simple wet bench procedures, and the ability to provide comprehensive testing results in a single assay. The emergence of WGS as a viable clinical testing platform has been made possible by advances over the past decade in methods for preparing and sequencing short-insert DNA libraries, which have made this process faster, more accurate, and less expensive. However, standard “short-read” sequencing (most often using 150 bp paired-end reads) has limitations, which have been shown by extension of the human reference genome to include more repetitive sequences^3^ and the growing catalog of complex genetic variants and disease-causing mutations.^4^, ^5^, ^6^ Recent studies have shown that sequencing of longer (generally >5 kbp), native DNA molecules with the Oxford Nanopore Technologies (ONT; Oxford, UK) and Pacific Biosciences (HiFi; Menlo Park, CA) platforms address these limitations and can improve detection and interpretation of complex variants and provide information about haplotype phase relationships across multiple sequence variants.^7^ Although use of the so-called “long-read” sequencing for routine clinical testing is still relatively uncommon,^8^ it has been used extensively in research studies to resolve complex structural rearrangements, and it is the primary technology used in ongoing efforts to improve and expand the human genome reference sequence.^3^^,^^9^^,^^10^

The success of these studies and continued advancements in long-read sequencing data production and analysis suggest that these platforms are poised to become competitive with standard short-read sequencing for clinical genomic testing. Although multiple reports have described methods and performance for detecting constitutional sequence variants from long-read data,^8^^,^^11^, ^12^, ^13^, ^14^, ^15^ less is known about the use of long reads for identifying somatic variants in cancer. The current study evaluated the potential for existing long-read sequencing platforms to be used for comprehensive genomic analysis of somatic sequence variants in AML and MDS. An automated workflow for data processing was developed, and a focused mutation analysis for clinically relevant somatic copy number variants (CNVs), SVs, and gene mutations, similar to the ChromoSeq approach developed for short-read WGS (sWGS), was performed.^2^ All samples tested in this study had matched sWGS analysis using our published method, allowing for head-to-head performance comparisons between long- and short-read platforms and examination of ways in which longer sequencing reads and phase-resolved alignments could add value to clinical genetic testing of myeloid neoplasms.

## Materials and Methods

### Patients and Samples

All samples were obtained from patients enrolled on an institutional tissue banking protocol and who provided written informed consent in accordance with the Declaration of Helsinki. Samples included peripheral blood or bone marrow collected before treatment that were either submitted directly for DNA extraction or processed buffy coat preparation and were cryopreserved. Supplemental Table S1 presents patient and sample details.

### Illumina WGS and Analysis

sWGS was performed on DNA extracted via the Qiagen DNA mini kit (Qiagen, Hilden, Germany) via the Illumina DNA library preparation kit and sequencing on NovaSeq 6000 or X Plus sequencers (Illumina). Sequence data processing, alignment, and somatic variant calling used DRAGEN version 4.2.4 (Illumina). Subsequent quality control, variant filtering for CNVs >2 Mbp, high-quality SVs, and small variants in 64 genes was performed as described previously.^2^ The hg38 reference sequence was used (complete with decoys and alternate contigs), and all annotations were based on Ensembl release 105 (*https://dec2021.archive.ensembl.org/index.html*, last accessed September 18, 2025).

### ONT and HiFi Sequencing and Data Processing

ONT sequencing was performed on DNA extracted and prepared as detailed in Supplemental Table S2. This process involved extraction kits for either standard or high molecular weight DNA and multiple approaches for fragmentation and size selection. Native DNA sequencing libraries were then constructed via ligation with the LSK114 kit and sequenced using R10.4.1 flowcells on a PromethION sequencer (Oxford Nanopore Technologies) with up to two wash/reload cycles. Data processing and analysis, including basecalling, were performed via a Nextflow workflow (*https://github.com/dhslab/nf-core-wgsnano*, commit 8fe4655) using dorado (version 0.5.2+7969fab) for basecalling and read mapping and the PEPPER-Margin-Deepvariant pipeline for phased germline variant calling of single nucleotide variants (SNVs) and small insertions/deletions (indels) (version 0.8.0).^16^ Haplotagging was performed using Whatshap (version 2.2).^17^ Mosdepth (version 0.3.3)^18^ was used to calculate coverage.

HiFi sequencing was performed via DNA extraction, fragmentation, size selection, and SMRTbell library preparation (Supplemental Table S3). Raw reads were analyzed on-instrument to produce circular consensus reads that were aligned by using pbmm2 (version 1.13.0). Germline SNV and small indels were called using DeepVariant (version 1.6.0),^19^ and phasing, haplotagging, and coverage analysis were performed the same as for ONT. All long-read data were mapped to the GRCh38 “no_alt_analysis_set” reference sequence recommended for long reads.

### Somatic Small Variant Analysis from Long Reads

Somatic SNV and indels were identified from the DeepVariant calls and using ClairS-TO (version 0.1.0)^20^ with the ‘ont_r10_dorado_5khz’ model for the ONT platform and the ‘hifi_revio’ model for the HiFi platform. Both the DeepVariant and ClairS-TO VCFs were annotated with Variant Effect Predictor^21^ using Ensembl release 105 to identify ‘nonsynonymous’ variants (namely, ‘splice_acceptor_variant,’ ‘splice_donor_variant,’ ‘stop_gained,’ ‘frameshift,’ ‘stop_lost,’ ‘start_lost,’ ‘transcript_ablation,’ ‘transcript_amplification,’ ‘inframe_insertion,’ ‘inframe_deletion,’ ‘missense_variant,’ and ‘protein_altering_variant’) in the set of 64 recurrently mutated AML genes; the transcript list is presented in Supplemental Table S4. Variants with maximum population minor allele frequency >0.1% were excluded, with the exception of specific hotspot mutations that were retained. The union of PASS variants from DeepVariant and PASS plus FILTER = ‘NonSomatic’ variants from ClairS-TO were taken to be the SNV/small indel callset.

### Somatic CNV and SV Analysis from Long Reads

Read-depth based CNVs were called using qdnaseq (version 1.34.0)^22^ in R version 4.2.3 (R Foundation for Statistical Computing, Vienna, Austria) with the following script (*https://github.com/epi2me-labs/wf-human-variation/blob/master/bin/run_qdnaseq.r*, commit 7295c35), using parameters *--binsize 100 --reference hg38 --cutoffLOSS 1.7 --cutoffGAIN 2.3*. Comparison of CNV calls was performed by using bedtools version 2.30.0.^23^ SVs were called by using Severus (version 0.1.1)^12^ and Sniffles2 (version 2.2) and restricted to rearrangements and deletions, insertions, or duplications >50 bp. Severus was run using default parameters and the provided bed file of VNTRs. Sniffles2 was run using parameters *--qc-coverage 0* and a bed file of tandem repeats. Svpack (*https://github.com/PacificBiosciences/svpack*, commit 9dddda6) was used for annotation with common SVs from two panels of healthy controls: the Human Pangenome Reference Consortium SV call set (*https://zenodo.org/records/8415406*, accessed June 5, 2024) and the Centers for Common Disease Genomics freeze1 SV callset.^4^ SV were annotated by using Variant Effect Predictor release 105 and then filtered for quality and predicted effect on AML-related genes (Supplemental Table S5).

All SVs were required to have a filter status of PASS (applied by Severus or Sniffles2), at least two supporting reads, and >5% abundance. Deletions, duplications, and insertions were required to be at least 5 kbp in length, within 20 kbp of a gene reported by the sWGS assay, and a “nonsynonymous” Variant Effect Predictor annotation consequence. Breakends (BND) and inversion calls were required to have either breakpoint within 20 kbp of any of 638 partner genes from 591 recurrent gene fusions in hematologic malignancies.^2^ Calls present in either the Human Pangenome Reference Consortium or Centers for Common Disease Genomics callsets were filtered out. SVs were classified as “recurrent” if both breakpoints occurred within 20 kbp of the two members of a recurrent gene pair. Filtered SVs were then clustered using minda (*https://github.com/KolmogorovLab/minda*, commit 3d35bba) to determine agreements between variants.

All manual review was performed by using Integrative Genomics Viewer version 2.16.0.^24^ Visualization of individual reads and variants was done by using Ribbon^25^ and SplitThreader.

## Results

### Long-Read Sequencing of Primary AML and MDS Samples

Long read sequencing with ONT and Pacific Biosciences circular consensus sequencing (HiFi) was used to characterize 26 primary blood or bone marrow samples from patients diagnosed with AML or MDS. Samples were obtained from patients with active disease who presented to our center and were either cryopreserved leukocytes from buffy coat prepared specimens or whole, unfractionated peripheral blood or bone marrow aspirate specimens. The cohort included patients with AML from all European LeukemiaNet risk categories and patients with MDS and high and low scores on the recently revised International Prognostic Scoring System (Supplemental Table S1). Ten samples were sequenced on both ONT and HiFi platforms for cross-platform comparisons. All samples were also tested using our published sWGS assay to identify small variants in 64 genes that are recurrently mutated in AML or MDS (Supplemental Table S4), large CNVs, and SVs predicted to affect genes (Supplemental Table S5); these were used as a gold standard for evaluation of the long-read platforms (Supplemental Tables S6, S7 and S8).

DNA preparation for long-read sequencing has yet to be standardized and can affect sequencing yield and quality. Several extraction approaches were evaluated in this study, including the DNA extraction method, DNA fragmentation, and DNA size selection to enrich for appropriately sized molecules (Supplemental Table S2 and Supplemental Figure S1). Between 2 and 7 μg of prepared DNA was then used to generate libraries for sequencing on one to five flowcells per SMRTcells per sample. For ONT sequencing, flowcells were run on the PromethION 24 instrument using 72-hour runtimes with up to two wash/reload cycles. All HiFi data were generated on a single SMRT Cell on the Revio instrument (Pacific Biosciences). Long-read data were processed by using a custom workflow that performed basecalling for ONT data (when not performed on-instrument), read mapping and alignment, and calculation of quality metrics. One advantage of the long-read platforms is the ability to phase reads into two haplotypes, given that the longer reads typically contain several heterozygous variants (on average 1 per kbp). The workflow therefore also performed automated germline variant calling, phasing, and haplotagging of individual reads with phase assignments (Supplemental Figure S2).

**Figure 1:**
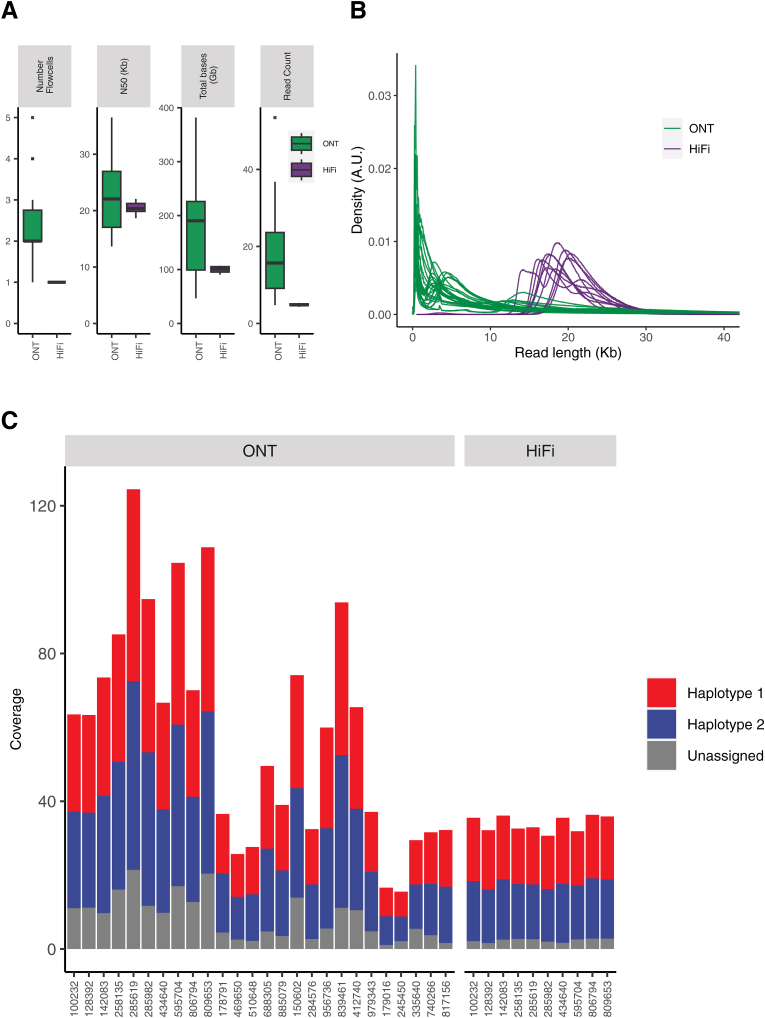
**A:** Sequencing yield and read metrics from Oxford Nanopore Technologies (ONT) and Pacific Biosciences HiFi sequencing platforms. **B:** Read length distributions for all samples from ONT and HiFi sequencing. **C:** Mean sequencing coverage per sample. Colors indicate coverage of haplotype 1 (red), haplotype 2 (blue), and no unassigned phase (orange). A.U., arbitrary units.

Sequencing yield and read statistics for all samples are shown in Figure 1A. Mean coverage for ONT samples was 59.0× compared with 34.0× for HiFi. Read lengths were evaluated for all libraries with respect to DNA preparation method and sequencing platform. DNA preparation for the HiFi platform was performed using a strict size-selection step that resulted in a tight distribution of read lengths around 20 kbp. Read lengths from the ONT platform were more variable due to the less-stringent size selection methods used before library preparation (Figure 1B and Supplemental Figure S1); however, N50 read lengths >15 kbp were observed in 24 (92%) of 26 samples. Most libraries used standard DNA extraction methods, indicating that high molecular weight extraction is not strictly necessary for this platform. Between 51% and 85% of mapped reads per sample could be phased (mean: 82.8% for HiFi; mean: 66.0% for ONT). The lower percentage of phased reads in the ONT samples is consistent with the higher proportion of shorter reads in those samples. Phased coverage ranged from 13.4× to 103.0× (mean, 50.5×) for the ONT samples and from 28.7× to 33.0× (mean, 31.6×) for the HiFi samples (Figure 1C). In terms of AML-specific genes targeted for focused mutation analysis (609 exons across 64 genes), the mean percentage of exons with at least 10× coverage for both haplotypes was 70.3%, although the range was large due to overall coverage differences between the samples.

### Targeted Somatic Variant Analysis from Long Reads

Variant calling from the ONT and HiFi data sets used a focused approach to emulate the sWGS assay. Because somatic variant calling from long-read data is an active area of development, several methods were tested before selecting those that gave the best overall performance for each variant type relative to sWGS in a nonblinded fashion. Multiple bioinformatics tools were ultimately used to make genome-wide variant calls that were then filtered for clinically relevant events (Materials and Methods). For SNV and indels, DeepVariant^16^ and ClairS-TO^26^ calls were used; qdnaseq^27^ was used for read-depth based CNV, and Severus^12^ and Sniffles2^13^ were used for breakpoint-mapped SVs (Supplemental Figure S2A). These calls were then filtered to retain large copy number alterations (>300 kbp), high-confidence genic SVs, and protein-altering SNV/small indel variants in recurrently mutated genes (Materials and Methods).

The specific genomic regions and variant filtering parameters were chosen to closely match those used for the sWGS assay to make the two data sets comparable for analysis. Using this approach, the total count of variant calls detected by the long-read platforms was similar to sWGS for all variant types (Supplemental Figure S2B and Supplemental Tables S9, S10, S11, and S12). Despite the differences in sequencing error rate across these technologies, a similar number of small variant calls in ONT, HiFi, and sWGS with means of 2.8 to 3.3 small variants per sample across platforms was also observed. For large copy number alterations, similar mean CNV counts (4.6, 6.3, and 11.4 for WGS, ONT, and HiFi, respectively) were observed. For SVs, a mean of 8.5, 15.5, and 22.6 events per sample for sWGS, HiFi, and ONT were recorded.

### Somatic SNV and Indel Performance Assessment

**Figure 2:**
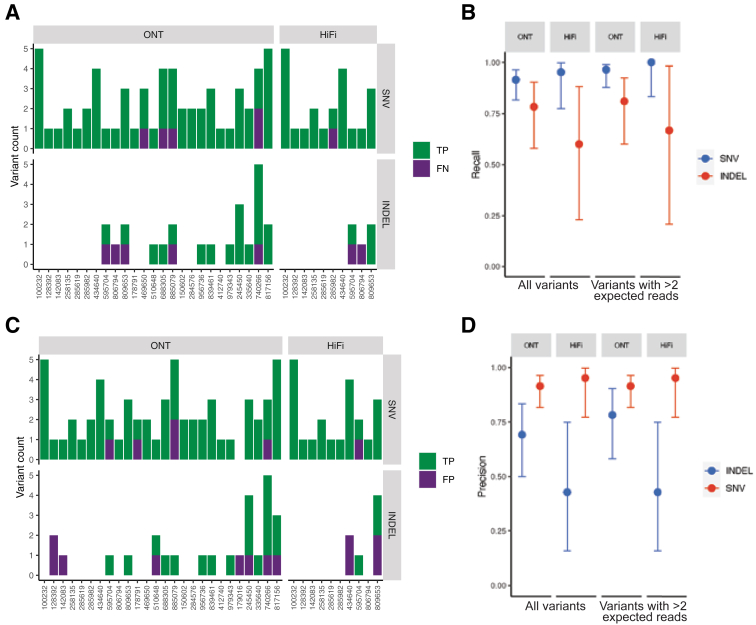
**A:** Small variants called by targeted analysis of short-read whole-genome sequencing (sWGS) in 59 genes. Colors indicate whether variants were detected via long-read sequencing (green) or in sWGS data only (purple). **B:** Recall for single nucleotide variants (SNVs) and small insertions/deletions (INDELS). Error bars indicate 95% Wilson score intervals. **C:** Small variants called by Oxford Nanopore Technologies (ONT) and Pacific Biosciences HiFi long-read sequencing. Colors indicate whether they were detected in sWGS (green) or in the long-read data only (purple). **D:** Precision for SNV and small INDELS. Error bars indicate 95% Wilson score intervals. FN, false negative; TP, true positive.

The accuracy of long-read SNV and indel variant calls was directly assessed by using the sWGS calls as a gold standard. For SNV with a variant allele fraction (VAF) >5%, recall rates were 91.5% (95% CI, 81.6%-96.3%) for ONT and 95.2% (95% CI, 77.3%-99.8%) for the HiFi data set. Recall rates for small indels of VAF >5% were 78.3% (95% CI, 58.1%-90.3%) for ONT and 60.0% (95% CI, 23.1%-88.2%) for HiFi (Figure 2, A and B). As expected, there was an increase in recall percentage for variants with higher VAFs, especially for samples in the long-read data set with lower coverage. Several of the variants were not detectable in the long-read samples due to a combination of low VAF and low coverage. Analysis was restricted to variants with adequate coverage (ie, an expected count of ≥3 variant reads given the sWGS VAF estimate and the long-read coverage; effectively, this excluded nine variants with median VAF of 11.8% and median long-read coverage of 21×). This resulted in higher recall rates: 96.4% SNV (95% CI, 87.9%-99.0%) and 81.0% indel (95% CI, 60.0%-92.3%) for ONT and 100% SNV (95% CI, 83.2%-100%) and 66.7% indel (95% CI, 20.7%-98.3%) for HiFi.

Next, the remaining seven false-negative variant calls in the long-read data, which included two SNVs and five indels, were reviewed. Of the two SNVs not detected by long reads, one was present in the long reads but at low VAF (Supplemental Figure S3A). The other discrepant SNV occurred in *U2AF1* and was missed due to a known error in the hg38 reference sequence^28^ that was not corrected in the reference version recommended for long-read analysis. Of the five indels missed by the long-read pipelines, one (in *KIT*) was clearly present at low VAF in the long reads (Supplemental Figure S3B), and a second (in *SRSF2*) was a trinucleotide variant that was called as an SNV by the long-read callers. The remaining three were all the same single-base frameshift insertion adjacent to a homopolymer repeat in *NF1*.

The precision for SNV was 91.5% for ONT (95% CI, 81.6%-96.3%) and 95.2% for HiFi (95% CI, 77.3%-99.8%) (Figure 2, C and D). For indels, the precision was lower for both platforms: 69.2% for ONT (95% CI, 50.0%-83.5%) and 42.9% for HiFi (95% CI, 15.8%-75.0%). Acknowledging that small indels are a common error mode in long-read variant calls, we implemented an additional filter (leveraging the ability to phase long reads) of excluding variants observed more than once in both haplotypes. This resulted in slightly improved precision for indels: 78.3% for ONT (95% CI, 58.1%-90.3%) and 42.9% for HiFi (95% CI, 15.8%-75.0%). After applying this filter, 15 false-positive small variants remained in the long reads: six SNVs (five in ONT, one in HiFi) and nine indels (five in ONT, four in HiFi). Manual review of the false-positive SNVs revealed that one was a partial representation of an multinucleotide variant in *SRSF2*, three were supported by a small number (between one and four) sWGS reads and may represent true low VAF variants, and two had no support in the sWGS data. None of the nine false-positive indel calls were supported by any high-quality sWGS reads. Notably, all false-positive indels occurred either on the X chromosome in male subjects (5 of 9 [55.6%]) or in regions with few phased reads in which phasing information could not be used for variant calling and filtration.

VAFs for both SNVs and indels showed good agreement between sWGS and the two long-read platforms (Supplemental Figure S4). Correlations in VAFs for SNV between sWGS and ONT and between sWGS and HiFi were 0.86 and 0.84, respectively. Similarly, for indels, correlations between sWGS and ONT and between sWGS and HiFi VAFs were 0.82 and 0.69.

### Phase-Informed Analysis of Small Variants in Long-Read Sequence Data

**Figure 3:**
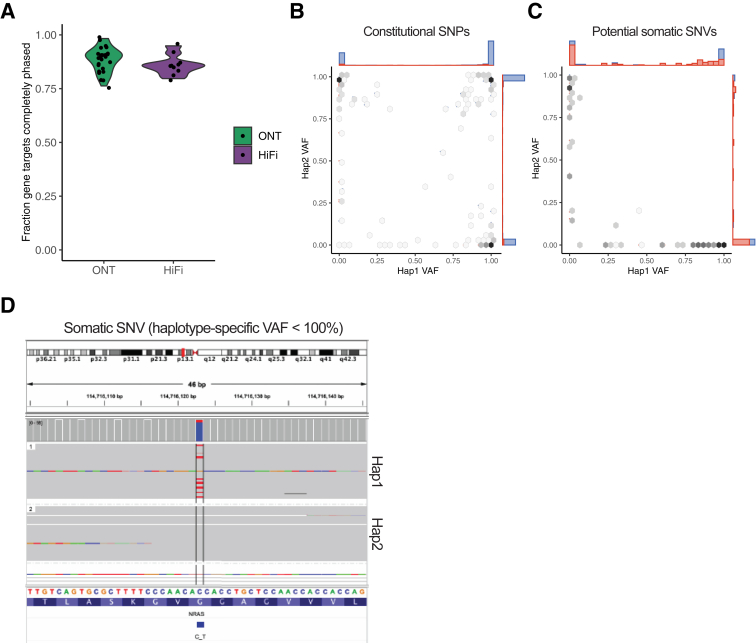
**A:** Fraction of completely phased gene targets per sample. **B:** Haplotype-specific variant allele fractions (VAFs) for reportable single nucleotide variants (SNVs) and small insertions/deletions (left) and unreported variants. Color of the marginal barplots indicate inferred somatic status: red, putative somatic; and blue, consistent with germline. **C:** Example somatic variant with haplotype 1 (Hap1) having a mixture of wild-type and mutant reads and a haplotype-specific VAF <100%. **D:** Example somatic SNV (NRAS c.38G>A) showing the allele is restricted to Hap1, which also has reads with the wild-type base, supporting its somatic status. Hap 1, haplotype 2; SNP, single nucleotide polymorphism.

Phase information provided by long reads has the potential to improve the interpretation of small variants by defining the relationship between variants in the same gene and distinguishing potential somatic variants from rare germline polymorphisms in tumor-only sequencing assays. This was investigated by first determining the number of targeted genes with complete and continuous phasing across their entire targeted lengths, which would enable direct assessment of *cis/trans* status for any identified variants. A mean of 51.8 of the 59 targeted genes (range, 45 to 58) per sample were completely phased (Figure 3A). Across our 26 samples, nine pairs of clinically relevant small variants occurring within the same gene were identified. Of these, eight pairs could be phased and thus definitively classified as occurring in *cis* or *trans*. Two variant pairs (in *RUNX1* and in *TET2*) were in *cis*, whereas six pairs (two in *TP53*, one in *NRAS*, and three in *TET2*) were in *trans* (Supplemental Figure S5).

Phased long reads can also help to determine the somatic status of a variant by the presence of “normal” reads (not containing the variant) within the “abnormal” haplotype in tumor samples with a clonal purity <100%. Restricting analysis to variants with >25× coverage, 110 (70.5%) of the 156 “reportable” SNVs and indels were classified as “putative somatic” (Materials and Methods). In contrast, examination of high-quality, clinically unreported variants occurring in the same targeted regions (ie, noncoding variants and variants with population frequency >0.1%) yielded only 60 (6.0%) of 1014 variants meeting criteria for classification as putative somatic (Figure 3, B–D). The remaining 94% of these variants had haplotype-specific VAFs consistent with germline heterozygous or homozygous mutations, showing the utility of this metric for *ad hoc* interpretation of variants of uncertain significance in cancer samples.

### Somatic Copy Number and SV Performance Assessment

We next compared CNVs and SVs identified using the long-read platforms with SV calls from sWGS. For CNVs, there was close agreement between large (>5 Mbp) read depth-based copy number aberrations. Genome-wide, an average of 120.4 Mbp per genome was affected by large-scale copy losses, and 78.8 Mb was affected by large-scale copy gains according to sWGS. Of the total chromosomal content called deleted by sWGS, 93.0% and 98.3% were called deleted in the ONT and HiFi data, respectively (Supplemental Figure S3A). Similarly, 87.6% and 94.7% of the copy gains by sWGS were called amplified in the ONT and HiFi data (Supplemental Figure S6). Manual review of the false-negative CNVs revealed that most were small (<2 Mbp) and/or had low abundances, suggesting they were subclonal events. Conversely, it was observed that nearly all the CNVs detected in the long-read samples were also detected by sWGS, with >95% of gains and losses identified by ONT and HiFi being present in the sWGS calls.

**Figure 4:**
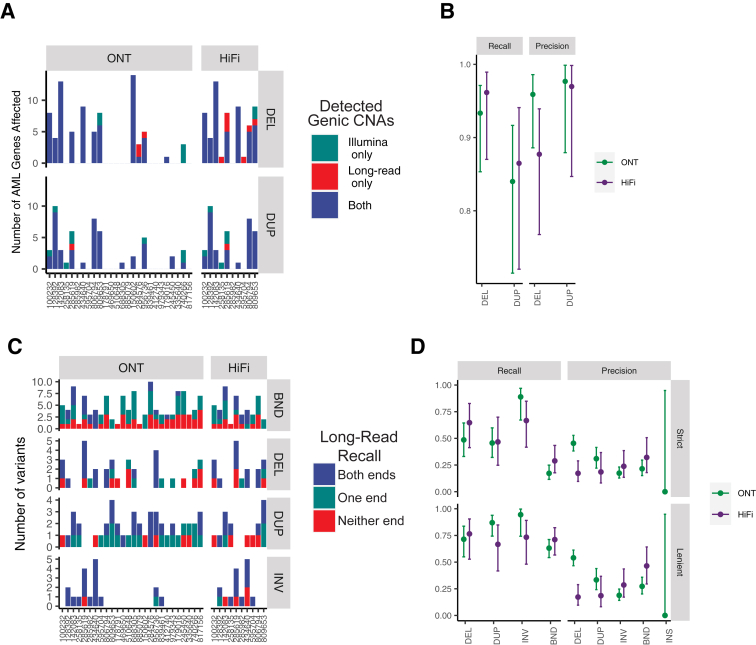
**A:** Counts of genes affected by read depth–based deletion (DEL) and duplication (DUP) events from Oxford Nanopore Technologies (ONT) (left) and Pacific Biosciences HiFi (right) platforms. Bars show the number of copy number variant (CNV)-affected genes, with colors indicating whether the CNV calls were present in short-read whole-genome sequencing (sWGS) only (green), long-read only (red), or both (blue). **B:** Recall and precision for gene-level CNV calls for ONT and HiFi using sWGS calls as the gold standard. **C:** Recall of reportable structural variants (SVs) called by the sWGS assay from long-read sequencing. Bars show the number of breakend-defined rearrangements (BND), deletions (DEL), duplications (DUP), and inversions (INV) from sWGS that were detected by either ONT or HiFi. Colors indicate whether the variant was supported by a long-read call on both (blue), one (green), or neither (red) of the BNDs. **D:** Recall and precision for BND-mapped SV, by SV type and platform. Panels indicate “strict” (top) versus “lenient” (bottom) definitions of overlap [strict overlaps with sWGS (ie, requiring overlap of both breakpoints), lenient overlaps (at least one breakpoint overlapping a sWGS call within 1 kbp)]. AML, acute myeloid leukemia; CNAs, copy number alterations.

A more clinically focused analysis of CNV calls between the platforms was also performed by comparing genes affected by copy number aberrations using the set of genes relevant to the diagnosis of myeloid malignancies that are reported by sWGS (Supplemental Tables S3 and S4). On average, 2.9 (range, 0 to 14) gene targets per sample were reported as deleted, and 1.9 (range, 0 to 10) AML gene targets per sample were reported as affected by copy gains by sWGS. For the long-read data sets, we detected 2.8 (range, 0 to 14) and 5.70 (range, 0 to 13) deletions per sample by ONT and HiFi, respectively, and similarly 1.7 (range, 0 to 9) and 3.3 (range, 0 to 9) amplifications per sample (Figure 4A).

Taking the sWGS calls as the gold standard, we observed recall rates of 93.3% (95% CI, 85.3%-96.1%) and 96.2% (95% CI, 87.0%-98.9%) for copy losses for ONT and HiFi, respectively, and, similarly, 84.0% (95% CI, 71.5%-91.7%) and 86.5% (95% CI, 72.0%-94.1%) recall rates for amplifications (Figure 4B). The positive predictive values for gene-level copy gains and losses were similarly high. For ONT, the precision values for gene-level deletion and amplification calls, respectively, were 95.9% (95% CI, 88.6%-98.6%) and 97.7% (95% CI, 87.9%-99.9%). For HiFi, the precision values for gene-level deletion and amplification calls were 87.7% (95% CI, 76.8%-93.9%) and 97.0% (95% CI, 84.7%-99.8%) (Figure 4B).

Discrepant calls were manually reviewed. Of the variants called by the sWGS pipeline but not the long reads, most were called at low VAF (<10%) and were not detectable by the long-read CNV callers, which were designed for germline variants. Of the variants called in the long reads but not the Illumina data, most were relatively small (<2 Mb) and were filtered out by the sWGS workflow.

To compare SV calls from the long-read data sets, the genome-wide long-read callset was filtered by using a strategy analogous to the one used for the sWGS assay. This approach restricted calls to recurrent driver SVs and those predicted to “impact” any of the targeted AML genes, after removal of constitutive SVs and artifacts from population-based SV studies.^4^ The recurrent driver SV calls is referred to as “recurrent SV” and the larger callset as “reportable SV,” given that they passed all filters and affect a targeted gene and thus may be of clinical or biological relevance. Considering only the recurrent SV category, the long-read platforms had 100% recall (of 12 events) and did not identify any additional recurrent SVs compared with sWGS across all 26 cases. Recall was lower for the reportable SV category; overall, we observed roughly two to three times the number of reportable SVs in the long-read SV callsets versus sWGS (Supplemental Figure S2B). Using a strict definition of recall that required both ends of an SV to coincide within 1 kbp, only 29.8% of reportable SVs from sWGS were supported at both breakpoints by a reportable SV detected in the long-read data (26.7% for ONT and 37.0% for HiFi). Recall was substantially higher when the comparisons were relaxed to require only one breakpoint to match [71.9% (95% CI, 65.7%-77.5%) for ONT; 71.7% (95% CI, 61.8%-79.9%) for HiFi] (Figure 4C), indicating some support in the long reads for these SV calls (see section on intronic insertions to follow).

**Figure 5:**
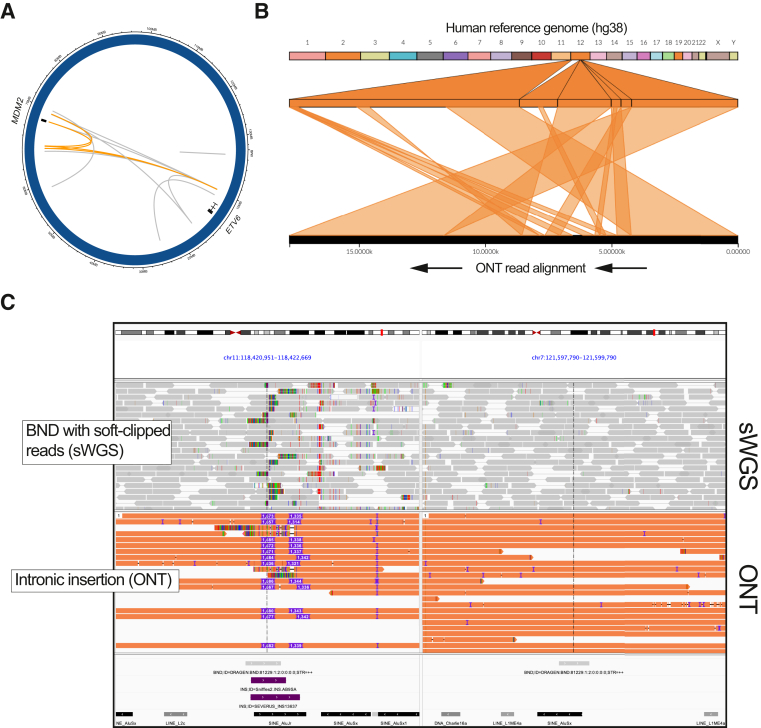
**A:** Complex rearrangement of chromosome 12 shown as a Circos plot, with arcs indicating novel adjacencies identified across the rearranged region. The **orange arcs** indicate breakpoints spanned by a single long read. **B:** Ribbon plot of the single long read spanning multiple breakpoints in the rearrangement depicted in **A**. **C:** A 1.6 kbp insertion at chr11:118.4 mol/L in ONT data (bottom track) that manifests as breakends (BND) in short-read whole-genome sequencing (sWGS) data. Note the sWGS has soft-clipped reads with the other end mapping to a distant region. ONT, Oxford Nanopore Technologies array.

Next, the precision for reportable SV calls from the long-read data was evaluated. The majority of these were not supported by a high-quality sWGS SV call (Supplemental Figure S7A). In terms of strict overlaps with sWGS (ie, requiring overlap of both breakpoints), the precision was 10.1% for ONT and 21.9% for HiFi. Considering lenient overlaps (at least one breakpoint overlapping a sWGS call within 1kbp), we observed precision of 33.0% (95% CI, 29.4%-37.0%) for ONT and 25.8% (95% CI, 19.6%-33.2%) for HiFi (Figure 4D). Review of the discrepant calls revealed that many (40%) occurred in complex and/or repetitive genomic regions (in particular, tandem repeats and segmental duplications), which are poorly covered by sWGS. Indeed, the precision for SVs in segmental duplications or simple tandem repeats was 1.0%, compared with 43.2% excluding those regions. Manual review of the discrepant variants in the long reads showed that most were high quality and simply not present in the sWGS callset due to poor mappability or differences in the filtering strategy between the two pipelines (Supplemental Figure S7B). Although none of the long-read-only SV calls would have changed the clinical interpretation of the sample tested, the ability of long reads to span some complex events provided a clearer view of their potential functional impact. For example, Figure 5A shows a complex SV involving multiple regions of chromosome 12 (near the AML genes *ETV6* and *MDM2*). Although parts of this complex event were detected by sWGS, long reads allow for direct visualization of the relationships between them (Figure 5B).

### Intronic Insertions Identified by Long-Read Sequencing Are a Source of False-Positive Structural Variants in sWGS Data

Given the high number of partially discordant SVs in the long-read data versus sWGS, further analysis was conducted to understand the nature of these discrepant events. As described earlier, discordant SV calls frequently had overlap with one breakpoint but not both. Further investigation of these events showed that 58% (63 of 109) of partially concordant reportable SVs were “BND” calls in sWGS that had one end overlapping an insertion in the long-read data (Figure 5C). Annotation of these insertions revealed that most of them coincided with insertions of mobile genetic repetitive elements that were not present in the GRCh38 human reference sequence. These events were not correctly resolved by sWGS and thus appeared as BND calls between distant (often interchromosomal) loci, which could be incorrectly interpreted as translocations. BNDs calls identified as insertions in the long-read data were a common source of such potential false-positive findings, accounting for 38.3% of all “gene-interrupting” BNDs in the reportable SVs from sWGS.

It was reasoned that the insights gained from paired long-read sequencing and sWGS of the same samples could improve the interpretation of intronic BND SV calls in sWGS. Therefore an additional filter was applied for SV calls from sWGS that excluded variants in which either end mapped within 2 kbp of a common insertion identified in the Human Pangenome Reference Consortium long-read callset.^29^ This is similar to the “systematic noise” filter for SVs that is already implemented in sWGS, except using source data from long-read sequencing rather than exclusively sWGS data. This additional filter excluded 78 (46.7%) of 167 nonrecurrent reportable SVs from the sWGS data; however, it did not remove any recurrent variants of known clinical significance, nor did it remove any variants supported on both ends by a reportable SV call from either of the long-read callers. This scenario suggests that use of variant databases from long-read data sets, applied strategically, can improve variant calls from sWGS.

## Discussion

This study includes a head-to-head comparison of WGS for somatic mutations in patients with AML and MDS using the ONT and HiFi assays versus a published sWGS assay, ChromoSeq. To simulate practical and realistic clinical testing procedures, long-read sequencing for most samples was performed by using standard DNA extraction techniques as opposed to high molecular weight extraction methods that are frequently used for long-read sequencing in research applications.

This analysis focused on small variants in a limited number of genes and clinically relevant CNVs and SVs that are reported by sWGS, which was previously shown to balance analytical performance with interpretability. Using this approach, it was found that both long-read platforms detected more than 96% and 81% of the SNVs and indels identified by sWGS, respectively. Because this study was not performed in a blinded fashion (ie, the sWGS results were used to guide the design of the automated variant calling pipelines), this represents an optimized performance of automated variant calling at the coverage depths achieved here. Most of the false-negative small variants in the long-read data set could be attributed to under-sampling in low coverage regions rather than systematic bias in either platform. Several false-positive small variants were observed in the long-read data, especially involving indels. Interestingly, these tended to occur in regions with inadequate phase information for both platforms, which is explicitly considered by the long-read variant callers. In the data obtained for this study (>20× total coverage and N50 read lengths of >13.6 kbp), we were able to phase 68% of all reads and produced contiguous phasing for a mean of 88% of the genes targeted for analysis in each sample. The phase information not only improved the accuracy of variant calls in these regions, but it was also useful for evaluating potential somatic variants and determining *cis/trans* relationships for somatic variants in eight genes. This underscores the importance of obtaining sufficient coverage of both haplotypes and the utility of performing this bioinformatics step to achieve optimal variant calling using long reads. It also shows ways in which phase information can be used bioinformatically and during case review to augment interpretation of existing variant quality metrics.

The benefits of long reads have been most apparent for SVs, in which multiple studies have reported better variant detection performance, especially in repetitive and low complexity regions. In our targeted SV calling approach, we observed 100% accuracy for recurrent SVs that are most clinically relevant in AML and MDS. Although long-read sequencing did not identify any clinically relevant results that were missed by sWGS, it did improve calls in repetitive regions, consistent with previous reports.^30^^,^^31^ The most notable new findings in the long-read data were partially concordant SVs that shared one breakpoint with the sWGS data. These were often annotated as unspecified BND calls by our sWGS assay that turned out to be medium-size insertions in the long-read data, typically involving nearby mobile element insertions. In many cases, the sWGS SVs could potentially be interpreted clinically as deleterious translocations involving other chromosomes or distant sequence partners, rather than insertions (often intronic) with less severe consequences. This information was used to apply an additional filter on the sWGS SVs that reduced these potential false-positive findings by almost 40%. This finding shows how long-read sequencing can increase the accuracy of current clinical genomic assays for cancer samples that use sWGS by establishing better rules and more comprehensive databases for interpreting SV calls produced by existing analysis pipelines.

A subset of the samples in this study were analyzed by both ONT and HiFi long-read sequencing, allowing for comparisons between the two platforms. A notable difference was the distribution of read lengths, which was highly variable in the ONT platform, owing to the less stringent DNA preparation methods. The HiFi data were more consistent but required greater input DNA amounts to obtain sufficient material for library preparation and sequencing, especially for samples with smaller average fragment sizes after extraction. Despite these differences, the fraction of phased reads was similar between the two platforms, indicating that both platforms yielded sufficient read lengths to capture heterozygous single nucleotide polymorphisms for phasing. Selection of one platform over the other for clinical testing may therefore depend on technical expertise or specific cost, throughput, or volume requirements, which do differ between the platforms (Supplemental Table S13).

In summary, this work shows that performance of long-read sequencing methods for the clinical evaluation of AML and MDS is comparable to that of sWGS for the detection of recurrent somatic SVs and CNVs. In addition, improvement in the accuracy of nonrecurrent SV calls in repetitive regions was observed. Although long-read sequencing allows for phasing, which can help distinguish between inherited and somatic variants, long-read somatic SNV and indel calling was less accurate than with sWGS. However, this could be improved with deeper coverage and further development of somatic variant calling methods, at which point these technologies would be an attractive approach for the clinical evaluation of AML and MDS.

## Disclosure Statement

D.H.S. and E.J.D have a pending patent and license agreement for ChromoSeq, a streamlined short-read sequencing assay for patients with AML and MDS that involves analysis methods similar (but not identical) to those described in this article.

## Supplemental Data

Supplemental Figure S1 Distribution of Oxford Nanopore Technologies (ONT) platform read lengths, by library. Panels indicate DNA extraction method. Line color indicates method of size selection. gDNA, genomic DNA; SFE, Short Fragment Eliminator.

Supplemental Figure S2 **A:** Overview of data processing approach for Oxford Nanopore Technologies (ONT) (left), Pacific Biosciences HiFi (middle), and Illumina (right) samples. **B:** Per-sample reportable variant counts by platform: top, single nucleotide variants (SNVs)/small insertions/deletions (indels); middle, read depth–based copy number variant (CNV); and bottom, structural variants (SV). BND, breakends; DEL, deletions; DUP, duplications; INS, insertions; INV, inversions.

Supplemental Figure S3 Examples of somatic variants identified by the short-read whole-genome sequencing (sWGS) pipeline and present, but not called, in the long reads. **A:** A single nucleotide variants (SNVs) in STAG2. **B:** A 6 bp in-frame insertion in KIT.

Supplemental Figure S4 Correlation in variant allele fraction (VAF) between single nucleotide variants (SNVs) (**blue**) and small insertions/deletions (INDELS) (**red**) called in the long-read data versus the Illumina data. Oxford Nanopore Technologies (ont) data are shown in the **left panel**, and Pacific Biosciences HiFi (pacbio) data in the **right panel**.

Supplemental Figure S5 Example showing two variants in *cis* in TP53, verified via multiple spanning long reads. Data shown are from Oxford Nanopore Technologies.

Supplemental Figure S6 Genome-wide agreement between short- and long-read read-depth based copy number variant (CNV) calls. Bar height indicates total genomic length affected by deletions (DEL) or duplications (DUP). Color indicates agreement between callers: blue, regions called in both short- and long-read assays; green, short-read only; and red, long-read only.

Supplemental Figure S7 **A:** Reportable structural variants called by the long-read workflows. Color indicates whether the variant was supported by short-read whole-genome sequencing (sWGS) on both, one, or zero breakpoints. **B:** Complex inversion called in the Oxford Nanopore Technologies (ONT) data but not by the Illumina pipeline. Both inversion breakpoints coincide with repetitive elements. BND, breakends; DEL, deletions; DUP, duplications; INS, insertions; INV, inversions.

## References

[1] Döhner H., Wei A.H., Appelbaum F.R., Craddock C., DiNardo C.D., Dombret H., Ebert B.L., Fenaux P., Godley L.A., Hasserjian R.P., Larson R.A., Levine R.L., Miyazaki Y., Niederwieser D., Ossenkoppele G., Röllig C., Sierra J., Stein E.M., Tallman M.S., Tien H.-F., Wang J., Wierzbowska A., Löwenberg B. (2022). Diagnosis and management of AML in adults: 2022 recommendations from an international expert panel on behalf of the ELN. Blood.

[2] Duncavage E.J., Schroeder M.C., O’Laughlin M., Wilson R., MacMillan S., Bohannon A., Kruchowski S., Garza J., Du F., Hughes A.E.O., Robinson J., Hughes E., Heath S.E., Baty J.D., Neidich J., Christopher M.J., Jacoby M.A., Uy G.L., Fulton R.S., Miller C.A., Payton J.E., Link D.C., Walter M.J., Westervelt P., DiPersio J.F., Ley T.J., Spencer D.H. (2021). Genome sequencing as an alternative to cytogenetic analysis in myeloid cancers. N Engl J Med.

[3] Nurk S., Koren S., Rhie A., Rautiainen M., Bzikadze A.V., Mikheenko A. (2022). The complete sequence of a human genome. Science.

[4] Abel H.J., Larson D.E., Regier A.A., Chiang C., Das I., Kanchi K.L. (2020). Mapping and characterization of structural variation in 17,795 human genomes. Nature.

[5] Chen S., Francioli L.C., Goodrich J.K., Collins R.L., Kanai M., Wang Q. (2024). A genomic mutational constraint map using variation in 76,156 human genomes. Nature.

[6] Gustafson J.A., Gibson S.B., Damaraju N., Zalusky M.P.G., Hoekzema K., Twesigomwe D. (2024). High-coverage nanopore sequencing of samples from the 1000 Genomes Project to build a comprehensive catalog of human genetic variation. Genome Res.

[7] Logsdon G.A., Vollger M.R., Eichler E.E. (2020). Long-read human genome sequencing and its applications. Nat Rev Genet.

[8] Paschal C.R., Zalusky M.P.G., Beck A.E., Gillentine M.A., Narayanan J., Damaraju N., Goffena J., Storz S.H.R., Miller D.E. (2025). Concordance of whole-genome long-read sequencing with standard clinical testing for Prader-Willi and Angelman syndromes. J Mol Diagn.

[9] Sirén J., Monlong J., Chang X., Novak A.M., Eizenga J.M., Markello C., Sibbesen J.A., Hickey G., Chang P.-C., Carroll A., Gupta N., Gabriel S., Blackwell T.W., Ratan A., Taylor K.D., Rich S.S., Rotter J.I., Haussler D., Garrison E., Paten B. (2021). Pangenomics enables genotyping of known structural variants in 5202 diverse genomes. Science.

[10] Jarvis E.D., Formenti G., Rhie A., Guarracino A., Yang C., Wood J. (2022). Semi-automated assembly of high-quality diploid human reference genomes. Nature.

[11] Vollger M.R., Logsdon G.A., Audano P.A., Sulovari A., Porubsky D., Peluso P., Wenger A.M., Concepcion G.T., Kronenberg Z.N., Munson K.M., Baker C., Sanders A.D., Spierings D.C.J., Lansdorp P.M., Surti U., Hunkapiller M.W., Eichler E.E. (2020). Improved assembly and variant detection of a haploid human genome using single-molecule, high-fidelity long reads. Ann Hum Genet.

[12] Keskus A.G., Bryant A., Ahmad T., Yoo B., Aganezov S., Goretsky A. (2025). Severus detects somatic structural variation and complex rearrangements in cancer genomes using long-read sequencing. Nat Biotechnol.

[13] Smolka M., Paulin L.F., Grochowski C.M., Horner D.W., Mahmoud M., Behera S., Kalef-Ezra E., Gandhi M., Hong K., Pehlivan D., Scholz S.W., Carvalho C.M.B., Proukakis C., Sedlazeck F.J. (2024). Detection of mosaic and population-level structural variants with Sniffles2. Nat Biotechnol.

[14] Jiang T., Liu Y., Jiang Y., Li J., Gao Y., Cui Z., Liu Y., Liu B., Wang Y. (2020). Long-read-based human genomic structural variation detection with cuteSV. Genome Biol.

[15] Geyer J., Opoku K.B., Lin J., Ramkissoon L., Mullighan C., Bhakta N., Alexander T.B., Wang J.R. (2025). Real-time genomic characterization of pediatric acute leukemia using adaptive sampling. Leukemia.

[16] Shafin K., Pesout T., Chang P.-C., Nattestad M., Kolesnikov A., Goel S., Baid G., Kolmogorov M., Eizenga J.M., Miga K.H., Carnevali P., Jain M., Carroll A., Paten B. (2021). Haplotype-aware variant calling with PEPPER-Margin-DeepVariant enables high accuracy in nanopore long-reads. Nat Methods.

[17] Martin M, Ebert P, Marschall T, Peters BA, Drmanac R (2022). Haplotyping: Methods and Protocols.

[18] Pedersen B.S., Quinlan A.R. (2017). Mosdepth: quick coverage calculation for genomes and exomes. Bioinformatics.

[19] Poplin R, Chang PC, Alexander D, Schwartz S, Colthurst T, Ku A, Newburger D, Dijamco J, Nguyen N, Afshar PT, Gross SS, Dorfman L, McLean CY, DePristo MA (2018). A universal SNP and small-indel variant caller using deep neural networks. Nat Biotechnol.

[20] Chen L., Zheng Z., Su J., Yu X., Wong A.O.K., Zhang J., Lee Y.L., Luo R. (2025). ClairS-TO: a deep-learning method for long-read tumor-only somatic small variant calling. bioRxiv.

[21] McLaren W., Gil L., Hunt S.E., Riat H.S., Ritchie G.R.S., Thormann A., Flicek P., Cunningham F. (2016). The ensembl variant effect predictor. Genome Biol.

[22] Scheinin I., Sie D., Bengtsson H., van de Wiel M.A., Olshen A.B., van Thuijl H.F., van Essen H.F., Eijk P.P., Rustenburg F., Meijer G.A., Reijneveld J.C., Wesseling P., Pinkel D., Albertson D.G., Ylstra B. (2014). DNA copy number analysis of fresh and formalin-fixed specimens by shallow whole-genome sequencing with identification and exclusion of problematic regions in the genome assembly. Genome Res.

[23] Quinlan A.R., Hall I.M. (2010). BEDTools: a flexible suite of utilities for comparing genomic features. Bioinformatics.

[24] Robinson J.T., Thorvaldsdóttir H., Winckler W., Guttman M., Lander E.S., Getz G., Mesirov J.P. (2011). Integrative genomics viewer. Nat Biotechnol.

[25] Nattestad M., Aboukhalil R., Chin C.-S., Schatz M.C. (2020). Ribbon: intuitive visualization for complex genomic variation. Bioinformatics.

[26] Zheng Z., Su J., Chen L., Lee Y.L., Lam T.W., Luo R. (2023). ClairS: a deep-learning method for long-read somatic small variant calling. bioRxiv.

[27] Sie D., Scheinin I., van Lieshout S., Cordes M., Pinkel D., Albertson D.G., Van de Wiel M., Ylstra B. (2016). Abstract 52: QDNAseq: a bioinformatics pipeline for DNA copy number analysis from shallow whole genome sequencing with noise levels near the probabilistic lower limit imposed by read counting. Clin Cancer Res.

[28] Miller C.A., Walker J.R., Jensen T.L., Hooper W.F., Fulton R.S., Painter J.S., Sekeres M.A., Ley T.J., Spencer D.H., Goll J.B., Walter M.J. (2022). Failure to detect mutations in U2AF1 due to changes in the GRCh38 reference sequence. J Mol Diagn.

[29] Liao W.-W., Asri M., Ebler J., Doerr D., Haukness M., Hickey G. (2023). A draft human pangenome reference. Nature.

[30] Chaisson M.J.P., Sanders A.D., Zhao X., Malhotra A., Porubsky D., Rausch T. (2019). Multi-platform discovery of haplotype-resolved structural variation in human genomes. Nat Commun.

[31] Zhao X., Collins R.L., Lee W.-P., Weber A.M., Jun Y., Zhu Q., Weisburd B., Huang Y., Audano P.A., Wang H., Walker M., Lowther C., Fu J., Gerstein M.B., Devine S.E., Marschall T., Korbel J.O., Eichler E.E., Chaisson M.J.P., Lee C., Mills R.E., Brand H., Talkowski M.E., Humane Genome Structural Variation Consortium (2021). Expectations and blind spots for structural variation detection from long-read assemblies and short-read genome sequencing technologies. Am J Hum Genet.

