# Supplementary figures and images for: Evaluation of Long-Read Genome Sequencing for Genomic Profiling of Myeloid Cancers
Source: J Mol Diagn. 2025 Sep 26;27(12):1242–54. doi: 10.1016/j.jmoldx.2025.09.001 (PMC12831091; doi:10.1016/j.jmoldx.2025.09.001)

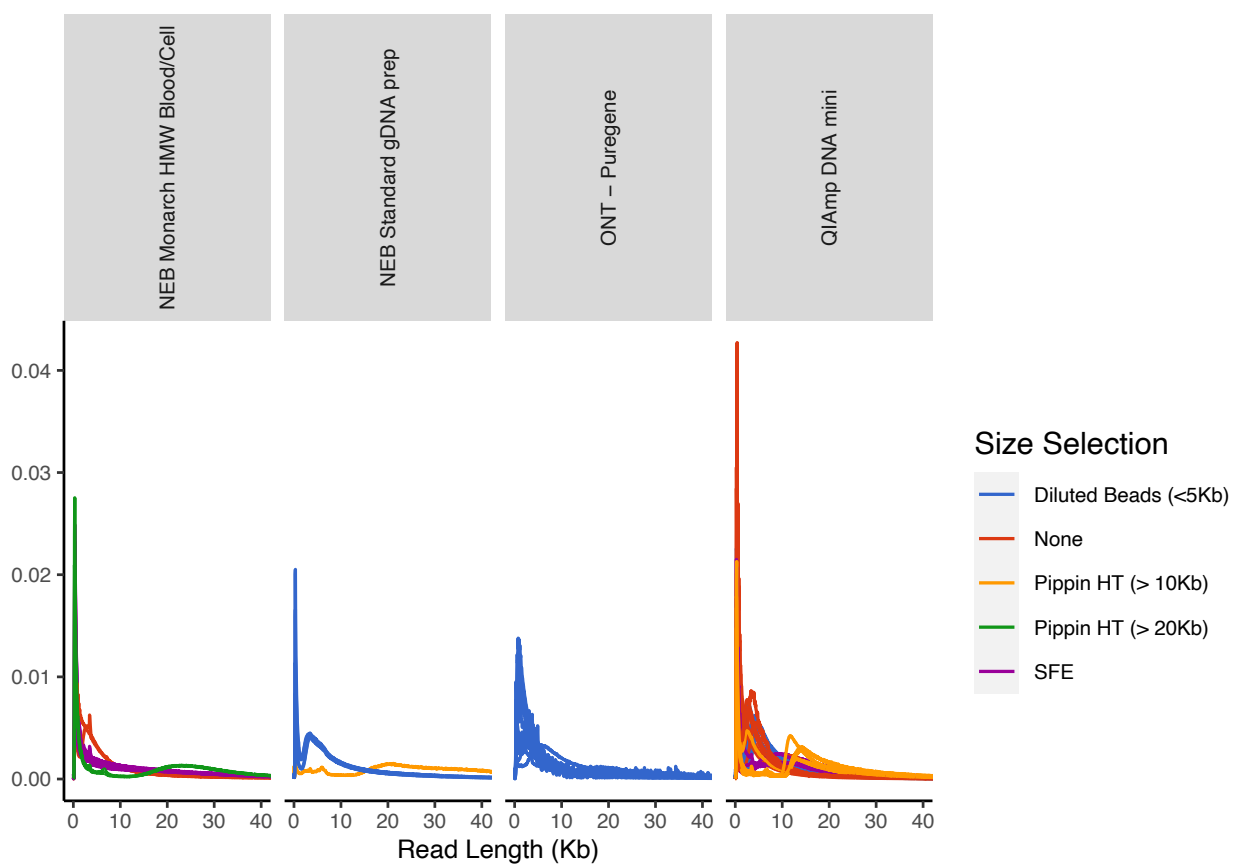

**Supplemental Figure S1**

Supplement: Supplemental Figure S1 — Distribution of Oxford Nanopore Technologies (ONT) platform read lengths, by library. Panels indicate DNA extraction method. Line color indicates method of size selection. gDNA, genomic DNA; SFE, Short Fragment Eliminator. [file mmc1.pdf]

A

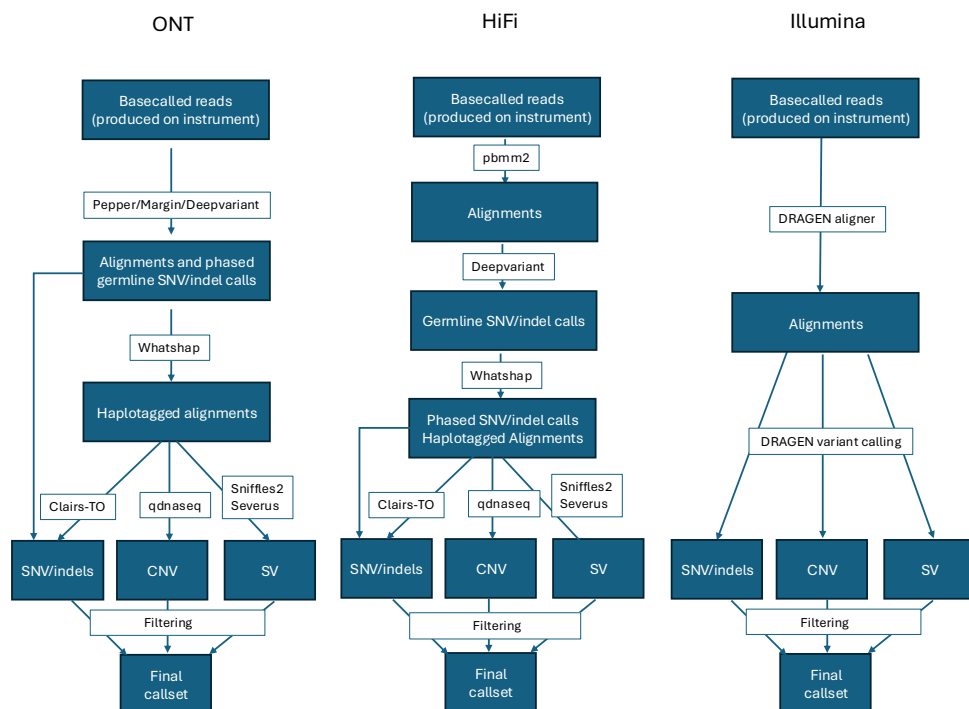

B

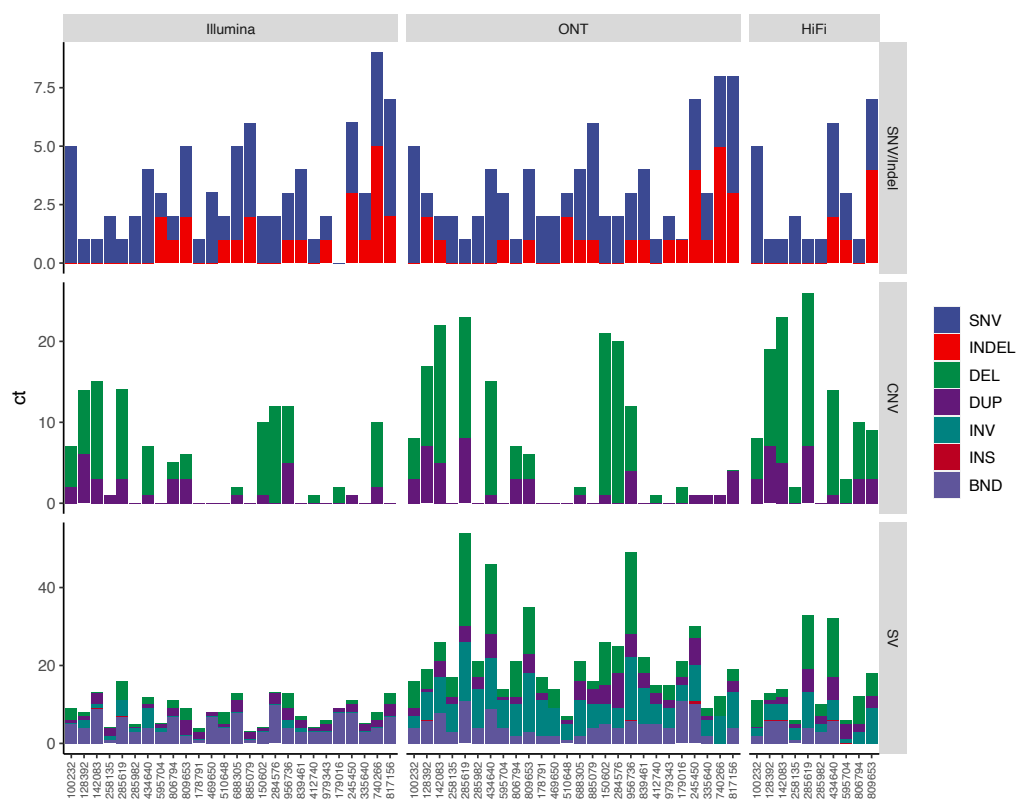

Supplemental Figure S2

Supplement: Supplemental Figure S2 — A: Overview of data processing approach for Oxford Nanopore Technologies (ONT) (left), Pacific Biosciences HiFi (middle), and Illumina (right) samples. B: Per-sample reportable variant counts by platform: top, single nucleotide variants (SNVs)/small insertions/deletions (indels); middle, read depth–based copy number variant (CNV); and bottom, structural variants (SV). BND, breakends; DEL, deletions; DUP, duplications; INS, insertions; INV, inversions. [file mmc2.pdf]

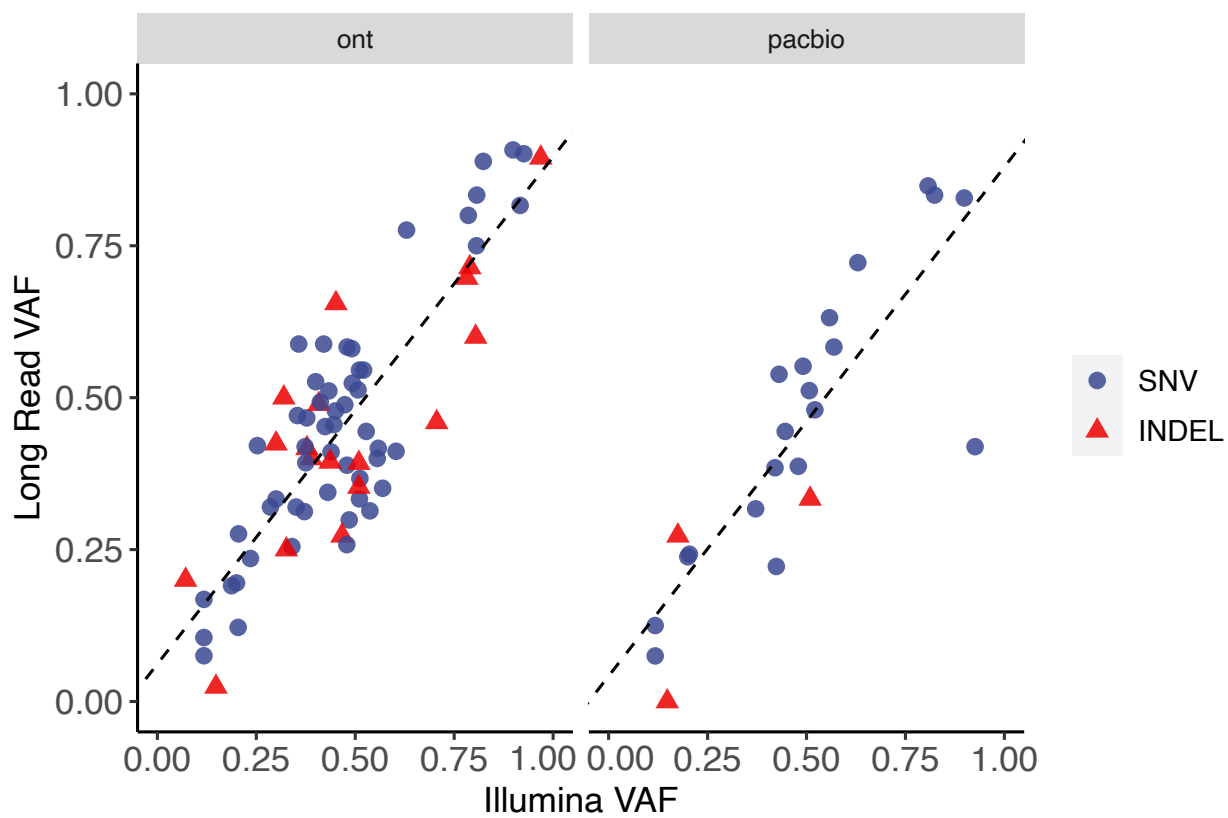

**Supplemental Figure S4**

Supplement: Supplemental Figure S4 — Correlation in variant allele fraction (VAF) between single nucleotide variants (SNVs) (blue) and small insertions/deletions (INDELS) (red) called in the long-read data versus the Illumina data. Oxford Nanopore Technologies (ont) data are shown in the left panel, and Pacific Biosciences HiFi (pacbio) data in the right panel. [file mmc4.pdf]

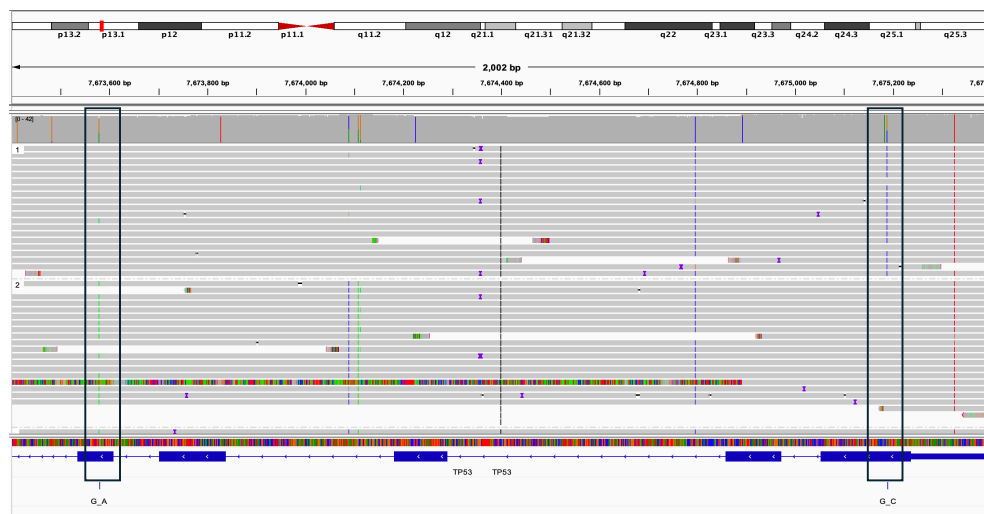

**Supplemental Figure S5**

Supplement: Supplemental Figure S5 — Example showing two variants in cis in TP53, verified via multiple spanning long reads. Data shown are from Oxford Nanopore Technologies. [file mmc5.pdf]

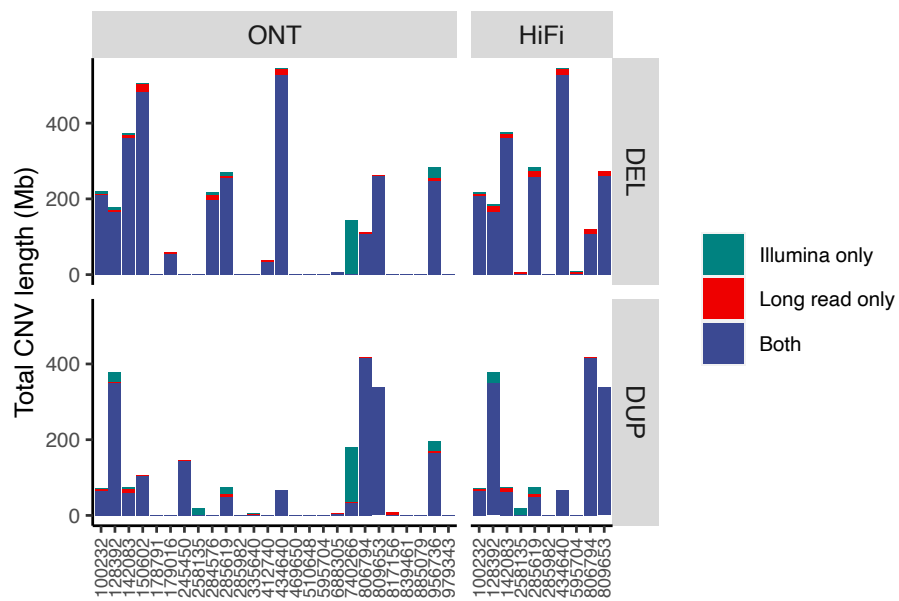

**Supplemental Figure S6**

Supplement: Supplemental Figure S6 — Genome-wide agreement between short- and long-read read-depth based copy number variant (CNV) calls. Bar height indicates total genomic length affected by deletions (DEL) or duplications (DUP). Color indicates agreement between callers: blue, regions called in both short- and long-read assays; green, short-read only; and red, long-read only. [file mmc6.pdf]

**A**

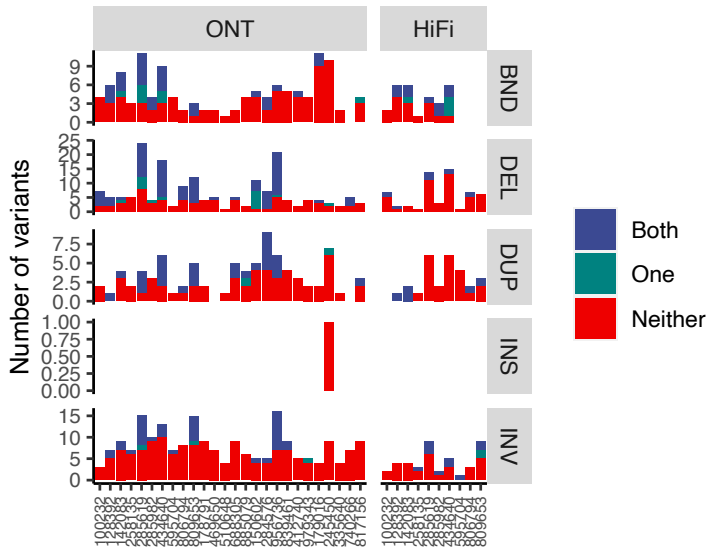

B

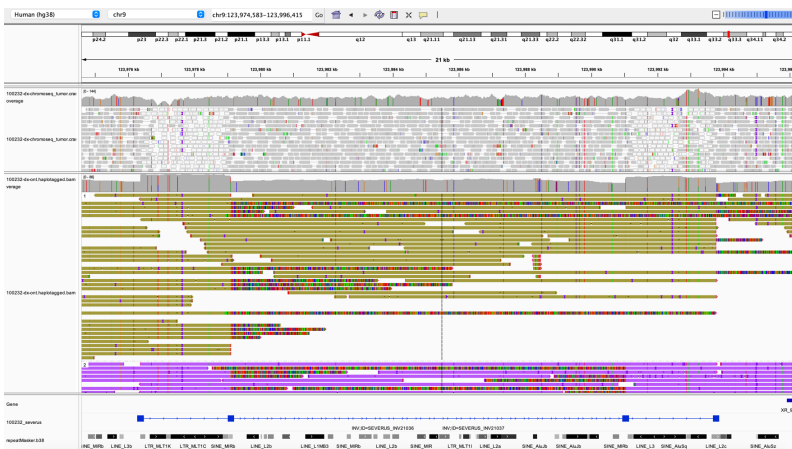

### Supplemental Figure S7

Supplement: Supplemental Figure S7 — A: Reportable structural variants called by the long-read workflows. Color indicates whether the variant was supported by short-read whole-genome sequencing (sWGS) on both, one, or zero breakpoints. B: Complex inversion called in the Oxford Nanopore Technologies (ONT) data but not by the Illumina pipeline. Both inversion breakpoints coincide with repetitive elements. BND, breakends; DEL, deletions; DUP, duplications; INS, insertions; INV, inversions. [file mmc7.pdf]
