# Supplementary material for: Evaluation of Long-Read Genome Sequencing for Genomic Profiling of Myeloid Cancers
Source: J Mol Diagn. 2025 Sep 26;27(12):1242–54. doi: 10.1016/j.jmoldx.2025.09.001 (PMC12831091; doi:10.1016/j.jmoldx.2025.09.001)
Supplement: Supplemental Figure S3 — Examples of somatic variants identified by the short-read whole-genome sequencing (sWGS) pipeline and present, but not called, in the long reads. A: A single nucleotide variants (SNVs) in STAG2. B: A 6 bp in-frame insertion in KIT. [file mmc3.pdf]

A

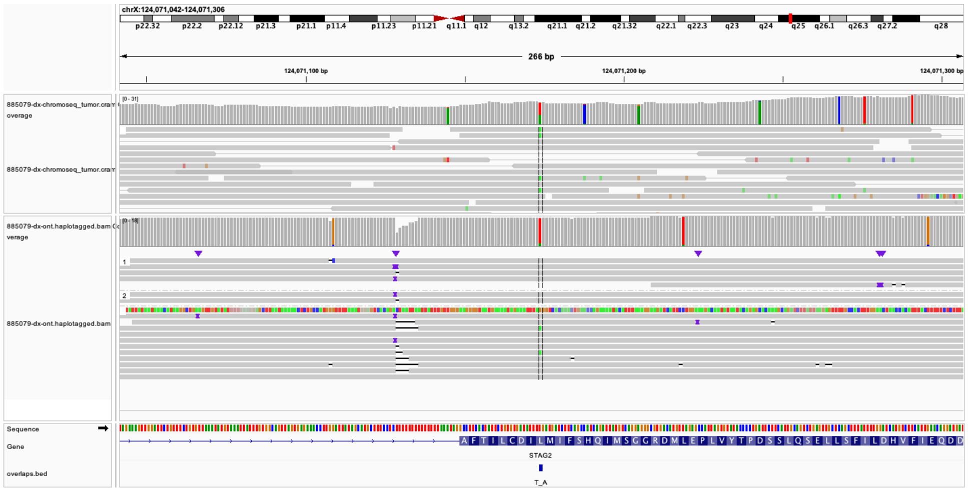

B

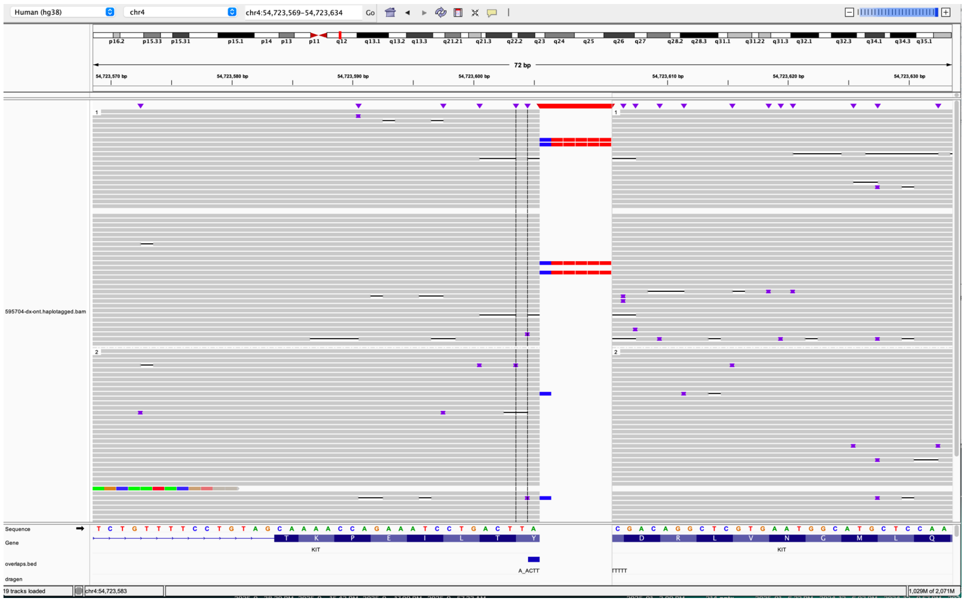

Supplemental Figure S3.
